# 

**Published:** 1889-06

**Authors:** 


### Your Correct Name.

If those members of the profession, who speak at the different dental conventions, will send us a postal card bearing their full name and titles we shall be much obliged, as we desire to make up a list for reference. In order to have your name appear in print as you want it, please attend to this at your earliest convenience.

### THOSE SUBSCRIPTION BILLS.

On the first of April we sent out bills to all persons who were in arrears for their journal. We have had returns from a large number, but not all. If any have forgotten to attend to the matter on account of the rush of work coming at this season of the year, let this be a gentle reminder, and attend to it before going away for the summer. We also desire to say to those, who had paid their subscription through some agent or depot, and who still received bills, that it was a mistake arising through crediting the house, and not the subscriber also, and which has been rectified. We are sorry to have bothered them, and will see that it does not occur again. Mistakes do sometimes occur in the best of regulated families, you know.

